# Morphological change of foveolar‐type gastric adenocarcinoma after proton pump inhibitor discontinuation in a short time period: A case report

**DOI:** 10.1002/deo2.293

**Published:** 2023-09-01

**Authors:** Shohei Igarashi, Norihiro Hanabata, Keisuke Furusawa, Shinya Suto, Miwa Satake, Koji Shimaya, Kosuke Kanazawa, Hiroshi Numao, Masaki Munakata, Hidekachi Kurotaki, Hirotake Sakuraba, Shigeaki Yoshida

**Affiliations:** ^1^ Department of Gastroenterology Aomori Prefectural Central Hospital Aomori Japan; ^2^ Department of Pathology Aomori Prefectural Central Hospital Aomori Japan; ^3^ Department of Gastroenterology and Hematology Hirosaki University Graduate School of Medicine Aomori Japan; ^4^ Aomori Prefectural Hospital Administration Aomori Japan

**Keywords:** endoscopic submucosal dissection, gastric cancer, immunohistochemistry, morphology, proton pump inhibitor

## Abstract

A 37‐year‐old man with systemic lupus erythematosus underwent esophagogastroduodenoscopy as a screening examination for anemia and bloody stool. A semi‐pedunculated edematous lobular polyp of 25 mm in size was detected in the greater curvature of the upper gastric body. At that time, a definitive diagnosis of cancer could not be made based on a biopsy specimen from the lesion. Since the patient was on proton pump inhibitor (PPI) for a long time to prevent peptic ulceration due to prolonged prednisolone administration for systemic lupus erythematosus, we diagnosed the lesion as a PPI‐associated hyperplastic polyp and switched lansoprazole to famotidine. Two months later, esophagogastroduodenoscopy revealed that the polyp had decreased in size to 8 mm, whereas the biopsy specimen led to a histological diagnosis of gastric cancer. The polyp was removed by endoscopic submucosal dissection. Immunohistochemistry revealed that the tumor cells were positive for MUC5AC, but negative for MUC2 and MUC6, leading to a final diagnosis of foveolar‐type gastric adenocarcinoma. In conclusion, we may suggest that PPI induces reversible morphological changes in foveolar‐type gastric adenocarcinoma. Furthermore, short‐term follow‐up of polypoid lesions should be prepared, considering tumor comorbidity with morphological changes during long‐term PPI usage.

## INTRODUCTION

Although foveolar‐type gastric adenocarcinoma (FTGA) has become widely known as one of the *Helicobacter pylori* (*HP)* non‐infected gastric cancers in recent years, the reports on FTGA are few and its clinicopathological characteristics remain unclear. Proton pump inhibitors (PPIs) induce various morphological changes in the gastric mucosa and modify the appearance of original polyps, such as gastric hyperplastic polyps (GHPs) or PPI‐associated hyperplastic polyps (PHPs). Consequently, PPI may make it difficult to distinguish between FTGA and other types of gastric polyps. To the best of our knowledge, there were no cases in which FTGA shrank after PPI discontinuation. Herein, we report a case showing drastic morphological changes in the FTGA briefly after PPI discontinuation.

## CASE REPORT

**FIGURE 1:**
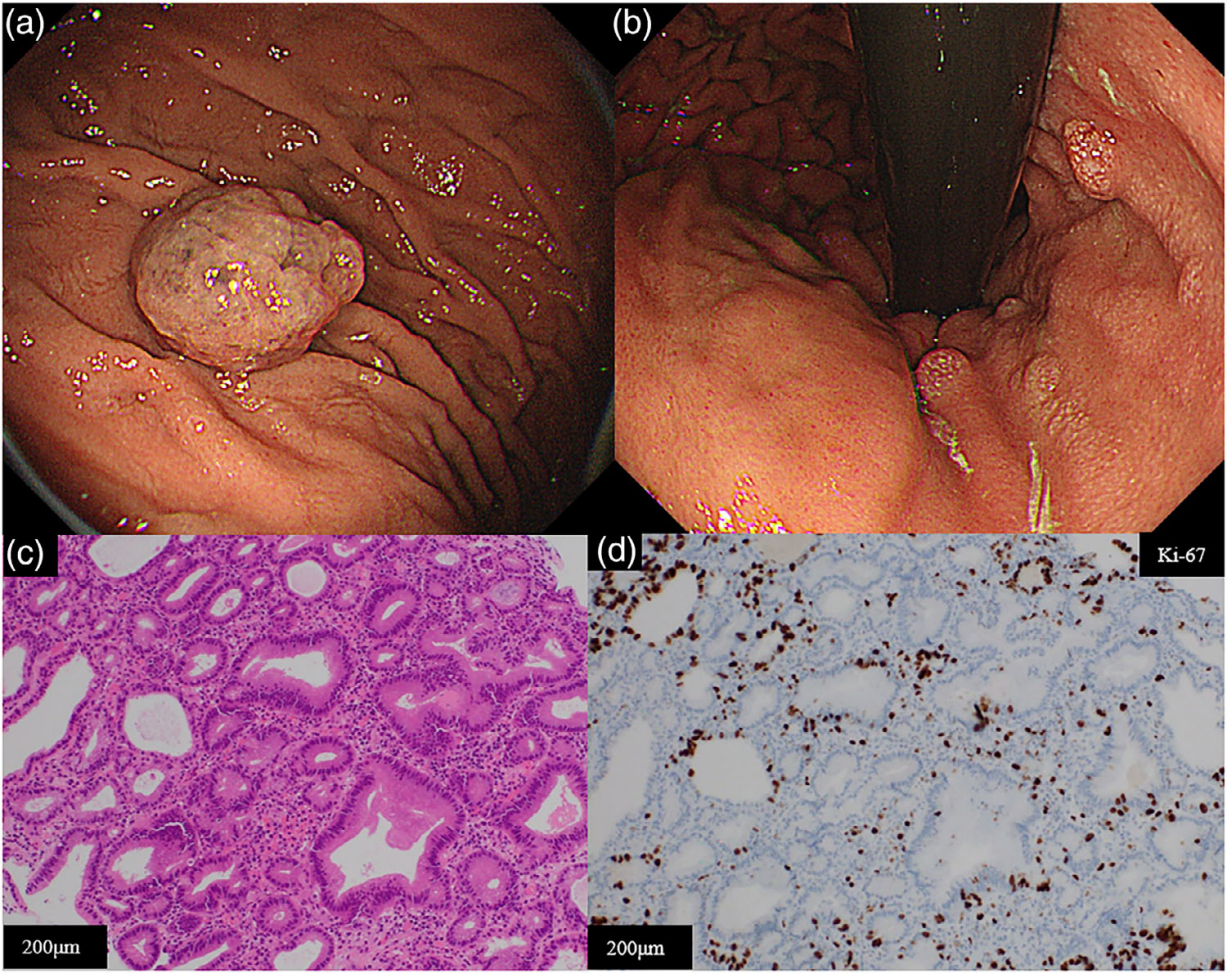
Endoscopic and histopathological findings of the first esophagogastroduodenoscopy. (a) White‐lite images show an edematous lobular semi‐pedunculated polyp: 25 mm in size. (b) Background gastric mucosa. There are multiple hyperplastic polyps sized <10 mm and whitish lanthanum deposits in the gastric mucosa. (c) Higher magnification of the biopsy specimen shows mild atypical glands (Hematoxylin and Eosin staining [HE]). (d) Ki‐67 labeling is focal in the specimen.

A 37‐year‐old man was referred to our hospital with bloody stools and anemia, which were suspected to be due to bleeding from colonic diverticulosis. He had been administered prednisolone for systemic lupus erythematosus and lansoprazole to prevent peptic ulcers for 10 years. The patient underwent esophagogastroduodenoscopy (to screen for anemia, which revealed no atrophic changes in the gastric mucosa, despite observing whitish lanthanum deposition. It was a result of his long‐term administration of lanthanum carbonate for hyperphosphatemia due to chronic kidney failure. In addition, an edematous lobular semi‐pedunculated polyp measuring 25 mm was observed on the greater curvature of the upper gastric body (Figure 1a). A malignant tumor was suspected due to the surface irregularity of the polyp. However, the two biopsy specimens from the top of the lesion were indefinite for neoplasia due to mild atypia and focally Ki‐67 labeling (Figure 1c,d). Multiple polyps, suggested as PHPs, were identified in the lesser curvature (Figure 1b). Biopsy specimens from these lesions revealed PHPs, which led to the diagnosis of PHPs.

**FIGURE 2:**
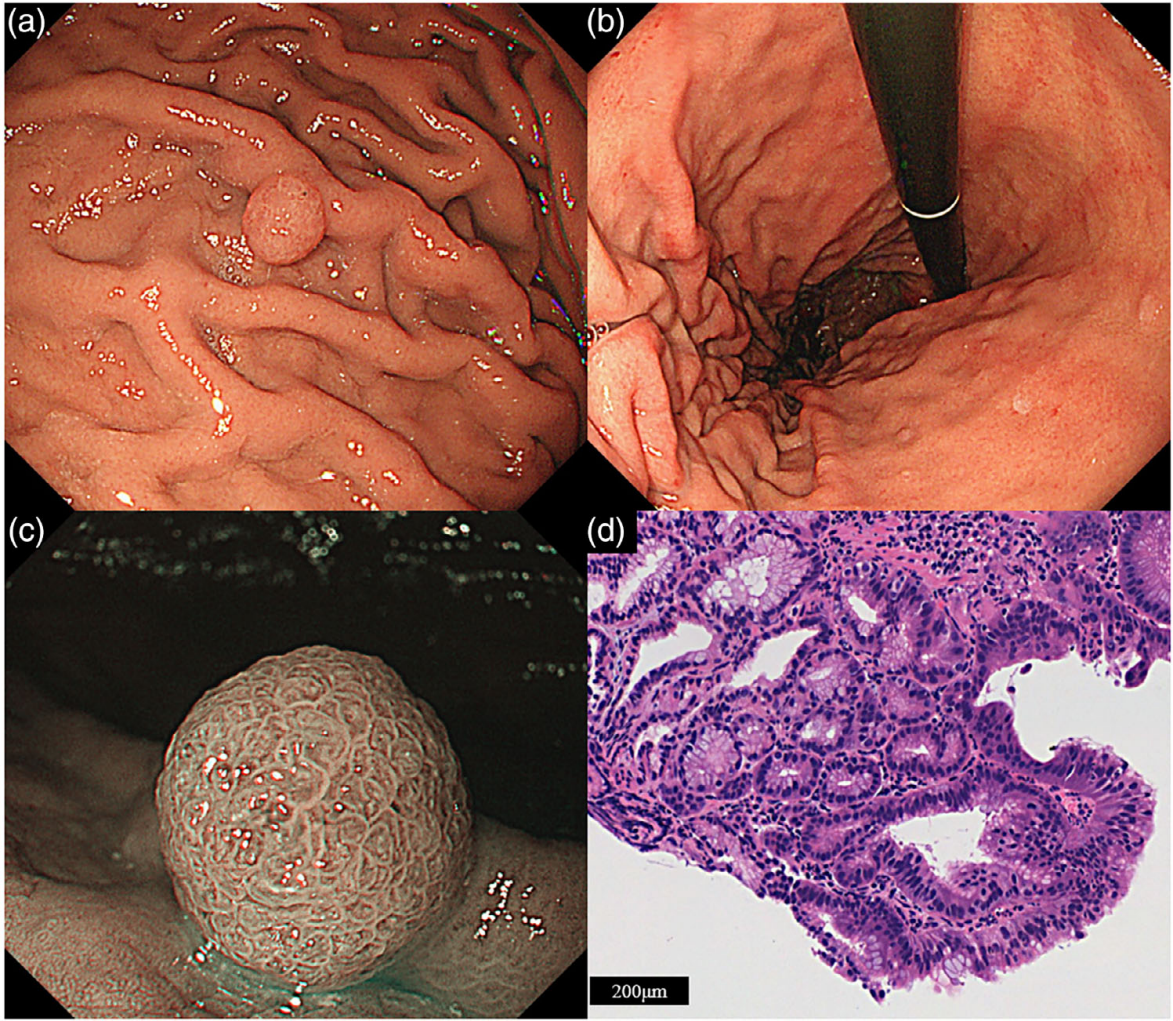
Endoscopic and histopathological findings after proton pump inhibitor (PPI) discontinuation. (a) The lesion shrinks in size to 8mm with a positive tone. (b) Background gastric mucosa. Multiple hyperplastic polyps shrink. (c) Magnified narrow‐band imaging combined with magnifying endoscopy reveals an irregular surface pattern after PPI discontinuation. (d) Higher magnification of the biopsy specimen shows structural and cellular atypia, which leads to the diagnosis of tubular adenocarcinoma (HE).

**FIGURE 3:**
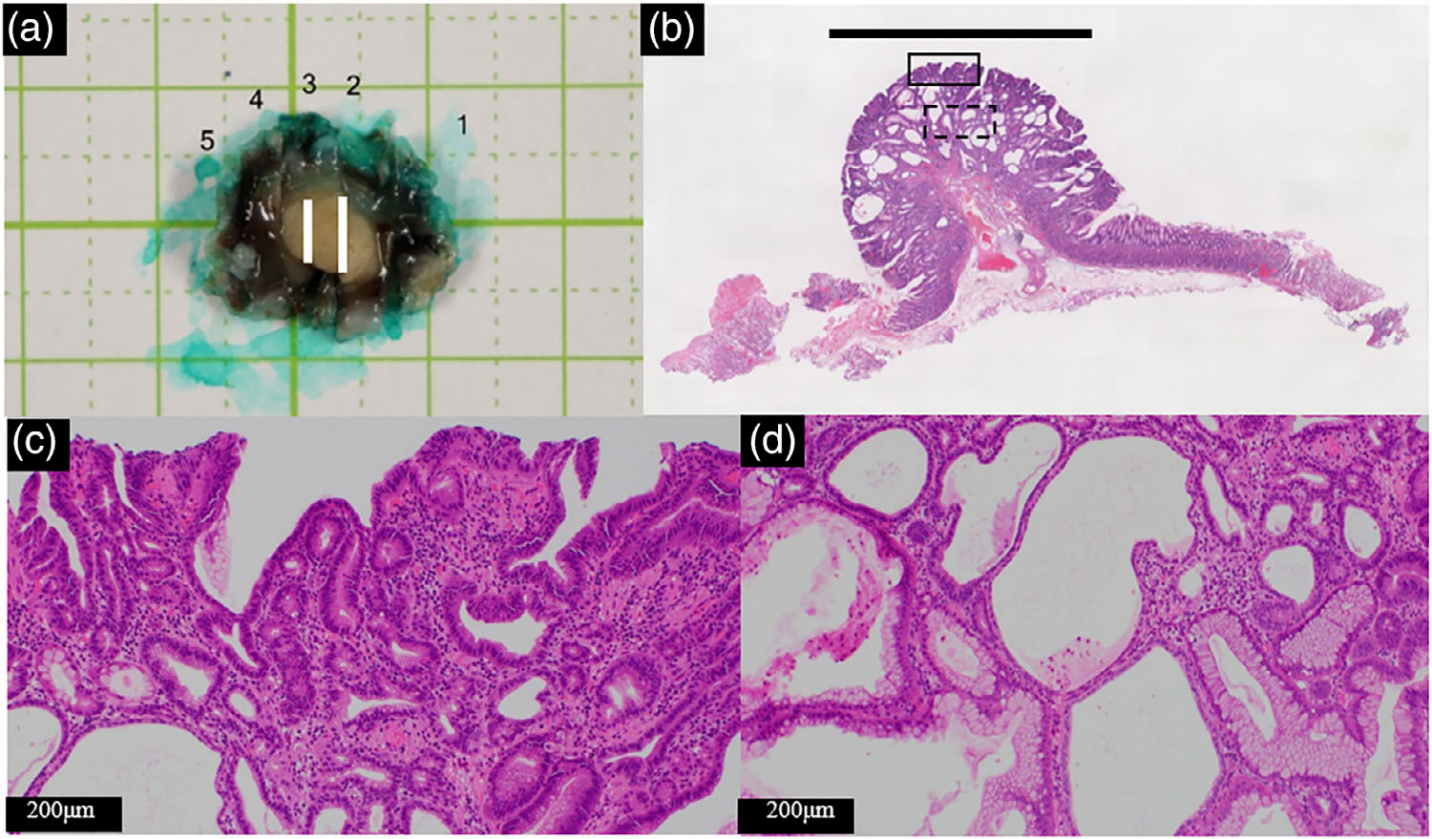
Histopathological findings of the endoscopic submucosal dissection specimen. (a) The specimen which is resected via endoscopic submucosal dissection (the white line shows the range of the lesion). (b) Histological images of the lesion in slice number 3. Hematoxylin–eosin staining. The black line above the tumor indicates the range of the lesion. (c) Higher magnification of the area of the black solid box shows the structural atypical tumor glands. (d) Higher magnification of the area of the black dashed box shows the dilation of fundic glands below the tumor cells.

**FIGURE 4:**
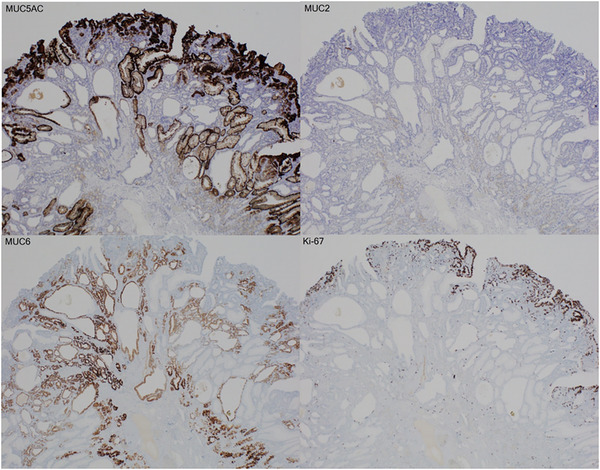
Immunohistochemistry findings. The tumor cells are positive for MUC5AC and negative for MUC2 and MUC6. Ki‐67 labeling is observed on the surface of the lesion.

Subsequently, we switched the patient's prescription from lansoprazole to famotidine and followed up with the polyps. The patient had no history of eradication, and *HP* infection was denied by the serum *HP* antibody levels (<5 pg/mL), fecal antigen of *HP*, and histopathological examination. Two months later, the polyp had decreased in size to 8 mm with a monotonous appearance (Figure 2a), and the other hyperplastic polyps disappeared (Figure 2b). Narrow‐band imaging combined with magnifying endoscopy (NBI‐ME) revealed structural irregularities on the surface of the lesion (Figure 2c). Additionally, biopsy specimens led to the pathological diagnosis of tubular adenocarcinoma (Figure 2d). The polyp was resected en bloc by endoscopic submucosal dissection (ESD) 2 months later. The polyp remained unchanged during this period. The histology of the ESD specimen was diagnosed with Type 0‐ I, 7×6 mm, Tubular adenocarcinoma, well‐differentiated, pT1a(M), pUL0, Ly0, V0, pHM0, and pVM0, based on the latest Japanese classification of gastric carcinoma. The lesion consisted of tubular adenocarcinoma in the surface layer (Figure 3a–c), manifesting cystic dilation of the fundic glands and hyperplasia of the foveolar epithelium (Figure 3d). Immunohistochemically, the tumor cells were positive for MUC5AC but negative for MUC2 and MUC6. Ki‐67 overexpression was observed in almost all tumor cells (Figure 4). The tumor cells were also negative for pepsinogen I and H+/K+ ATPase. The final pathological diagnosis was FTGA. The esophagogastroduodenoscopy performed 1 year after the ESD revealed no recurrence of gastric cancer or multiple hyperplastic polyps.

## DISCUSSION

Herein, we report a rare case of FTGA with morphological changes coupled with shrinkage after PPI discontinuation. Long‐term PPI administration may affect the morphology of FTGA. Therefore, after PPI discontinuation, short‐term follow‐up endoscopy may be an option for differentiating PHPs from those with malignancy, which is difficult to make, because polyp shrinkage may lead to detailed observation of its surface structure.

FTGA is one of the *HP*‐negative gastric cancers, considered a low atypical tumor, and frequently occurs in the upper/middle sections of the stomach, whereas it is similar to fundic gland polyps or GHPs, both morphologically and in site distribution. Thus, differential diagnosis of these lesions is important because their treatments differ, whether or not the polyp has malignant potential. NBI‐ME is useful for the diagnosis of FTGA, as it reveals papillary or gyrus‐like surface structures of varying shapes and irregularly dilated microcapillaries with dense growth.^1^ However, a method for distinguishing malignancy from these polyps has not yet been established even using the NBI‐ME. Consequently, pathology and immunohistochemistry are of utmost reliability. Immunochemistry shows FTGA‐tumor cells are positive for MUC5AC, whereas MUC2 is rarely observed and MUC6‐positive cells may be seen at the bottom of the glands.^2^ Initially, an accurate diagnosis of the polyp could not be made. We assumed that the reason why the initial biopsy resulted in an indefinite diagnosis of gastric cancer was attributable to the specimen taken just from a low atypical area in the tumor, which was focally Ki‐67 labeling in the ESD specimen. Consequently, as shown in our case, the shrinkage of the polyp following PPI withdrawal may lead to more detailed endoscopic observation and precise biopsy.

It is well‐known that PPIs could cause changes in the endoscopic appearance of the stomach in the form of side reactions such as fundic gland polyps, GHPs, and PHPs.^3^ Regarding the endoscopic findings of PHPs, they were reported as reddish, smooth, and dome‐shaped polyps <10 mm in diameter.^4^ On the contrary, some studies made reference to a case of a >20‐mm‐sized polyp in a non‐*HP* infected stomach and the difficulty of diagnosing neoplasia endoscopically even when using NBI‐ME.^4^ The reduction in the GHPs after the discontinuation of long‐term PPI usage is also well‐known.^5^ PPI withdrawal may be useful in patients with symptomatic large gastric polyps. However, we often hesitate to follow up or perform resection, if the patient has no symptoms and malignancy cannot be ruled out in the polyp. The size criterion (>25 mm) as a risk factor for neoplastic changes may be helpful.^6^ However, ESD for the lesion located in the greater curvature of the upper gastric body is technically difficult and highly invasive. Endoscopic mucosal resection is an effective procedure, by which en bloc resection of large lesions could be difficult. The polyp reduction following PPI discontinuation may make these procedures easier and safer.

PPIs are also considered risk factors for gastric cancer.^7^ Some reports described the development of FTGA during maintenance therapy with vonoprazan for reflux esophagitis.^8^ This might be concerning because FTGA could be one of the significant outcomes of long‐term antacid use. Thus, it is necessary to be aware of the development of tumors during prolonged PPI administration. In the present case, we suggest that the original FTGA was affected by PPI administration, considering the endoscopic findings after polyp shrinkage, although it might be difficult to prove rigorously due to the lack of previous endoscopic images before PPI initiation.

Long‐term PPI administration pathologically induces parietal cell protrusion, cystic dilation of the fundic glands, stromal edema, and foveolar epithelial hyperplasia.^9^ These histological changes can increase gastric polyps. In our case, the ESD specimen showed cystic dilation of the fundic gland below the tumor glands. However, parietal cell protrusion or stromal edema was not observed, as commonly observed in long‐term PPI users. It has been reported that parietal cell protrusion occurs in most patients within the first months after PPI treatment.^9^ Although there was no report on how long after PPI discontinuation these histological changes improved, some studies reported decreases in PHPs 1 month after.^10^ Thus, PPI withdrawal may have led to these reversible changes in a few months and contributed to tumor shrinkage and morphological changes. As with other factors, the collision of FTGA and PHP, or the impact of biopsy may have contributed to tumor shrinkage. However, collisional lesions were denied by almost even distribution of tumor cells in the ESD specimens. The impact of the biopsy was also difficult to consider because of not a small lesion and only two small biopsy samples from the surface of the polyp.

In conclusion, our case suggests that long‐term PPI administration may induce reversible morphological changes in FTGA. Therefore, it is imperative to follow up on the possibility of cancer comorbidities in any gastric polyps during PPI usage.

## CONFLICT OF INTEREST STATEMENT

None.
